# A conserved axon type hierarchy governing peripheral nerve assembly

**DOI:** 10.1242/dev.106211

**Published:** 2014-05

**Authors:** Liang Wang, Alessandro Mongera, Dario Bonanomi, Lukas Cyganek, Samuel L. Pfaff, Christiane Nüsslein-Volhard, Till Marquardt

**Affiliations:** 1 Developmental Neurobiology Laboratory, European Neuroscience Institute (ENI-G), Grisebachstraße 5, Göttingen 37077, Germany; 2 Department of Genetics, Max-Planck Institute for Developmental Biology, Spemannstrasse 35, Tübingen 72076, Germany; 3 Gene Expression Laboratories, Howard Hughes Medical Institute, The Salk Institute of Biological Studies, 10010 N. Torrey Pines Road, La Jolla, CA 92037, USA

**Keywords:** Axon guidance, Axon-axon interactions, Motor neurons, Peripheral nerve, Sensory neurons, Sympathetic neurons, Mouse, Chick, Zebrafish

## Abstract

In gnathostome vertebrates, including fish, birds and mammals, peripheral nerves link nervous system, body and immediate environment by integrating efferent pathways controlling movement apparatus or organ function and afferent pathways underlying somatosensation. Several lines of evidence suggest that peripheral nerve assembly involves instructive interactions between efferent and afferent axon types, but conflicting findings challenge this view. Using genetic modeling in zebrafish, chick and mouse we uncover here a conserved hierarchy of axon type-dependent extension and selective fasciculation events that govern peripheral nerve assembly, which recapitulates the successive phylogenetic emergence of peripheral axon types and circuits in the vertebrate lineage.

## INTRODUCTION

Nervous system evolution seems to have frequently proceeded via the use of pre-existing axon pathways, rather than through *de novo* formation of nerve tracts, to accommodate novel features within extant circuits (Katz, 1983; Katz et al., 1983). The segmental organization of the vertebrate body axis, for example, places constraints on peripheral axon growth that force primary somatosensory axons (SAs) to extend through peripheral nerve tracts that are also occupied by more ancestral motor efferent axons (MEs) (Bonanomi and Pfaff, 2010; Butler and Hodos, 2005). Such co-confinement to narrow substrate corridors may effectively foster interactions between axons that extend from phylogenetically newer or older neuron types, and accelerate their incorporation into common functional assemblies.

Indeed, peripheral nerve assembly has long been thought to involve prerequisite association of SAs with earlier extending MEs en route to peripheral targets (Hamburger, 1929; Taylor, 1944; Honig et al., 1986; Swanson and Lewis, 1986; Honig et al., 1998). For example, surgical or laser-mediated removal of ventral neural tube segments, including motor neurons, in amphibian and avian embryos was observed to frequently result in the development of aneural limb muscle (Hamburger, 1929; Honig et al., 1986; Swanson and Lewis, 1986; Taylor, 1944). Our own recent data provided a mechanistic basis for some of these ideas by showing that, in mouse, SAs are guided to targets in the dorsal trunk by molecular labels on earlier extending epaxial MEs (Wang et al., 2011). Genetic manipulations that completely blocked ME extension resulted in randomized extension of SAs along dorsal or ventral trajectories, whereas ME-restricted elimination of the EphA receptor tyrosine kinases EphA3 and EphA4 triggered selective dorsal-to-ventral misrouting of SAs (Wang et al., 2011; Wang and Marquardt, 2013).

Other recent data on mouse limb innervation, however, support a minimal cooperative model according to which mutual interactions between MEs and SAs have only a limited influence on the establishment of peripheral nerve trajectories (Huettl et al., 2011). For example, upon partial genetically induced elimination of MEs in mouse embryos, only mild SA extension defects were observed, whereas elimination of DRG neurons or DRG neuron-derived neuropilin receptor expression led to pronounced defasciculation, but not to mistargeting, of MEs (Huettl et al., 2011). Studies based on surgical manipulations in frog and chick embryos arrived at yet different conclusions, proposing that establishment of limb SA and ME trajectories can be entirely dissociated from each other (Wang and Scott, 1999; Wenner and Frank, 1995). Consolidating these conflicting lines of evidence remains difficult because differences in animal models, methodologies and positional identities of the peripheral nerve segments studied currently preclude their direct comparison.

## RESULTS

### Conserved reliance of sensory axon extension on pioneer motor axons

**Fig. 1.**
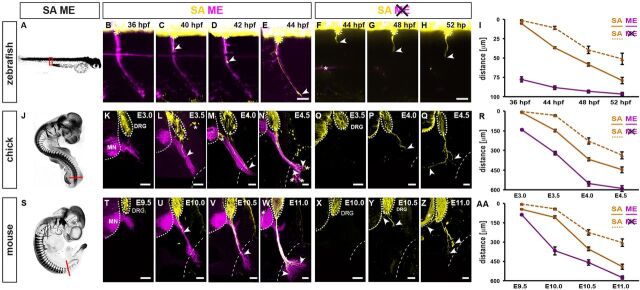
**Conserved reliance of sensory afferent axon (SA) extension on pioneer motor efferent axons (MEs).** (A) Peripheral axons visualized in 72 hpf zebrafish larva. (B-E) Time-lapse sequence of SA, labeled using *-8.4neurog1::GFP* transgene (yellow), extending peripherally along preformed CaP ME, labeled by *NBT::dsRed* transgene (magenta). Arrowheads indicate SA growth cone. (F-H) Delayed and aberrant SA extension in the absence of MEs upon *islet1E2/E3* morpholino injection. (I) Longitudinal analysis of SA and ME extension and impacts of ME removal (*n*≥7 segments, ≥3 embryos per stage and condition). (J) Peripheral axons visualized in E4 chick embryo. (K-N) SAs, labeled using the *Isl1^DRG^::mGFP* transgene (yellow), extending along preformed MEs, labeled by co-transfected *Hb9^MN^::mCherry*. Arrowheads indicate distalmost SA growth cone. Asterisks indicate additional *Isl1^DRG^::mGFP* activity in non-DRG neural crest cells. Dotted line indicates trunk/limb boundary. (O-Q) Delayed and aberrant SA extension (visualized using anti-Tuj1 antibody) in the absence of MEs upon *Hb9^MN^*:*:Cre*/*PGKneolox2DTA* co-transfection. (R) Longitudinal analysis of relative SA and ME extension, and impact of ME removal (*n*≥7 sections, ≥3 embryos per stage and condition). (S) Peripheral axons visualized in E10.5 mouse embryo. (T-W) SAs, labeled using the *Brn3a^tau:lacZ^* allele, extending along preformed MEs, labeled using the *Hb9^MN^::GFP* transgene. (X-Z) Delayed and aberrant SA extension in the absence of MEs in *Olig2^Cre^;Rosa26^lxstopDTA^* embryos. (AA) Longitudinal analysis of relative SA and ME extension, and impact of ME removal (*n*≥8 sections, ≥4 embryos per stage and condition). hpf, hours post-fertilization; DRG, dorsal root ganglion; MN, motor neurons. Error bars indicate standard error of the mean (s.e.m.). Scale bars: 10 μm in E,H for B-H; 50 μm in K-Q,T-Z.

To explore the interactions between primary somatosensory afferent axons (SAs) and motor efferent axons (MEs) we first systematically investigated the relationships between molecularly identified peripheral axon types in three different vertebrate species: zebrafish (*D. rerio*: Fig. 1A), chick (*G. gallus domesticus*: Fig. 1J) and mouse (*M. musculus*: Fig. 1S). In anamniotes, including zebrafish, the first emerging sensory-motor circuits are dedicated to simple larval escape reflexes that are mediated by an early central nervous system (CNS) neuron population, Rohon Beard cells (RBs), which feed primary sensory inputs from dermis to motor neurons controlling trunk musculature (Spitzer, 1984). RBs are eventually replaced by neural crest-derived dorsal root ganglion (DRG) neurons that become incorporated into circuits facilitating a wider spectrum of motor outputs (Butler and Hodos, 2005; Dasen, 2009; Holland, 2009). In zebrafish, these circuit rearrangements are reflected by a ∼20 h delay between initiation of primary ME extension, visualized by a motor neuron-specific regulatory module of the *NBT* (*Xenopus* β-tubulin) gene to drive red fluorescent protein expression (dsRed) (Peri and Nusslein-Volhard, 2008), and the emergence of SAs from DRGs, visualized by exploiting the DRG neuron-restricted activity of the neurogelin 1 promoter to drive green fluorescent protein (GFP) expression (Blader et al., 2003) (Fig. 1B-E; supplementary material Fig. S1A-E). SAs thus invariably extended along preformed ME trajectories. 

To visualize SAs and MEs in chick, we used previously identified enhancer modules of the *Isl1* and *Hb9* genes (Lee et al., 2004) to confine green and red fluorescent protein expression to DRG and motor neurons, respectively (supplementary material Fig. S2A-F). In mouse, DRG and motor neurons axons were respectively visualized by the previously established DRG and motor neuron-specific transgenes *Brn3a^tau:lacZ^* and *Hb9::eGFP* (Lee et al., 2004; Trieu et al., 2003). In amniotes, including chick and mouse, formation of larval Rohon Beard circuits is skipped, and primary sensory-motor circuits directly assemble from motor neurons and DRG neurons (Dasen, 2009; Holland, 2009). Despite this altered configuration, the principal chronological sequence of peripheral axon extension observed in zebrafish appears to be preserved in chick and mouse: SAs invariably extended along trajectories pioneered by MEs, as reported previously (Fig. 1K-N,T-W; supplementary material Fig. S2A-F) (Honig et al., 1986; Wang et al., 2011).

We next tested whether this rigid axon type-dependent extension order would reflect a conserved primacy of the first-extending MEs during peripheral nerve assembly. To address this, we studied SA extension upon preventing ME extension. In zebrafish, this was achieved by injecting morpholino oligonucleotides targeting the *Isl1* gene that were previously found to selectively interfere with motor neurogenesis (Hutchinson and Eisen, 2006). The resulting absence of MEs led to severely reduced initial extension rates of SAs (Fig. 1F-I) and frequently triggered highly erratic patterns of SA extension (supplementary material Fig. S1L-Q). In chick, the prevention of ME extension, by introducing *Hb9-*driven cell-autonomous diphtheria toxin (DTA) into the neural tube prior to motor neurogenesis, resulted in a randomized loss of dorsal or ventral trunk SA pathways within the confines of normal peripheral nerve trajectories (supplementary material Fig. S2G-J), resembling those previously reported by us upon DTA-mediated ablation of motor neuron progenitors in mouse (Wang et al., 2011).

**Fig. 2.**
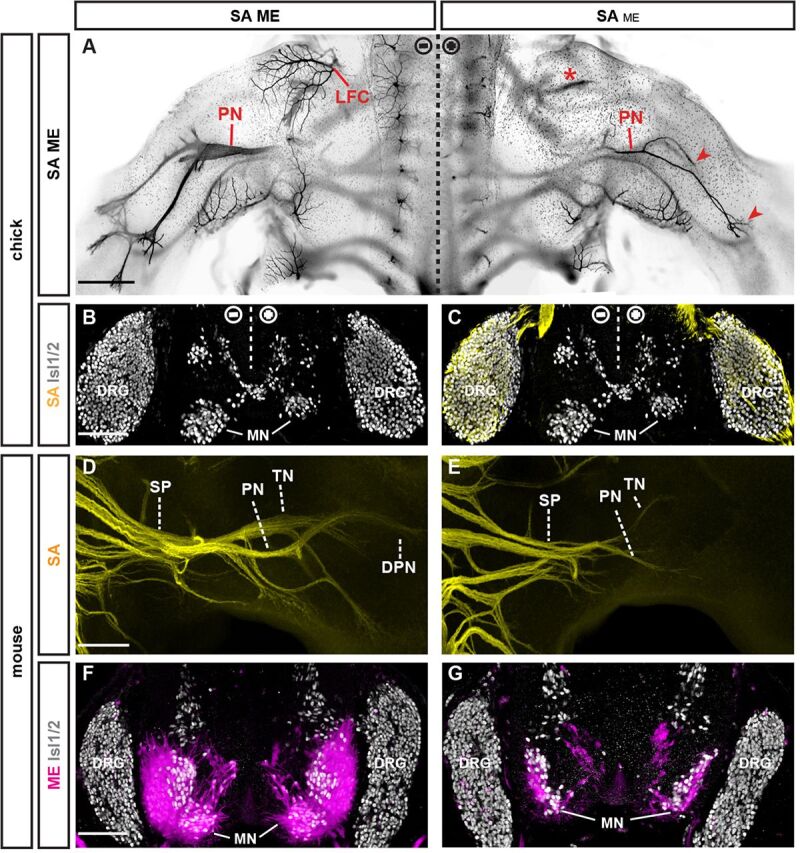
**Effects of partial ME ablation on SA extension.** (A) Dorsal whole-mount view of lumbar spinal cord and limbs in E6 chick embryo: peripheral axons visualized by anti-Tuj1 immunodetection (black). Severe reduction, but not loss, of crural (asterisk), peroneal (PN) and tibial nerves (TN) (arrowheads) upon unilateral transfection with a low titer (0.5 μg/ml) of *Hb9^MN^::Cre/PGKneolox2DTA* plasmids. LFC, lateral femoral cutaneous nerve. (B,C) Transverse section of E6 chick spinal cord: partial ablation of motor neurons (MNs) after unilateral low-titer transfection. Anti-Isl1/2 immunofluorescence (gray) to label DRG neurons and MNs. Anti-TrkA immunofluorescence (yellow) to label DRG neurons. (D) Dorsal whole-mount view of SAs (yellow indicates *Brn3a^tlz^*) at the sciatic plexus (SP) in E12.5 mouse embryo. DPN, deep peroneal nerve; PN, peroneal nerve; TN, tibial nerve. (E) Reduction, but not loss, of SAs beyond the sciatic plexus after delayed ablation of MEs in *Olig2^Cre^;Isl2^lxstopDTA^* embryo. (F) Transverse section of E12.5 control spinal cord: MNs labeled using *Hb9^MN^::GFP* (magenta). Anti-Isl1/2 immunofluorescence (gray) visualizes nuclei of DRG neurons and MNs. (G) Transverse section of E12.5 *Olig2^Cre^;Isl2^lxstopDTA^* spinal cord: severe reduction, but not complete absence, of MNs. Scale bars: 300 μm in A; 100 μm in B; 200 μm in D,F.

We next asked whether the apparently conserved role of MEs in establishing trunk SA trajectories would similarly govern assembly of peripheral nerve pathways that co-evolved with the tetrapod limb (Butler and Hodos, 2005; Luria et al., 2008; Ma et al., 2010). Similar to zebrafish, genetic ablation of motor neurons or their progenitors prior to ME extension in chick and mouse (supplementary material Figs S2G,H and S3A-J) resulted in markedly reduced SA extension rates (Fig. 1O-R,X-AA). In contrast to the effects of ME removal on chick and mouse trunk innervation, where SAs projected in a randomized manner within largely normal peripheral pathways (see supplementary material Fig. S2I,J) (Wang et al., 2011), lumbar SAs exhibited highly aberrant projections that frequently deviated from the trajectories normally chosen by peripheral axons (Fig. 1Q,Y,Z). These defects culminated in the failure or severe delay of most SAs to extend beyond the limb plexus (Fig. 1Q,R,Z,AA; supplementary material Fig. S3K,L), possibly owing to the markedly delayed SA extension. Under conditions of delayed or partial prevention of ME extension, however, impacts on the establishment of peripheral SA trajectories were considerably milder in both chick (Fig. 2A-C) and mouse (Fig. 2D-G), resembling results obtained by previous studies relying on late-stage surgical or incomplete genetic removal of motor neurons (Huettl et al., 2011; Wang and Scott, 1999; Wenner and Frank, 1995). 

### Sensory axons are invariably guided by motor axons

**Fig. 3.**
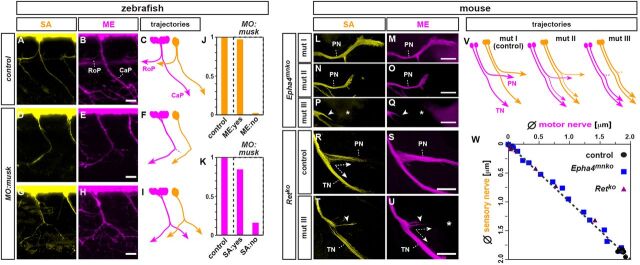
**SA trajectories are configured by pre-extending MEs.** (A-I) RoP and CaP SA (yellow) and ME (magenta) trajectories in 72 hpf control zebrafish embryos (A-C) and upon *unp-SV1-MO* morpholino injection (D-I). (A,B) Same principal trajectories followed by SAs and MEs in control embryo. (C) Reconstructed main ME and SA trajectories in control (staggered rendering of MEs and SAs for simplicity). (D-I) Examples of SAs (D,G) following aberrant ME trajectories, skipping trajectories devoid of MEs (E,H) in *unp-SV1-MO*-injected embryos. (F,I) Reconstructed main ME and SA trajectories of the same embryos. (J,K) Cumulative semi-quantification: ratio of nerve segments with SAs (J) or MEs (K) possessing (yes) or lacking (no) MEs (J) or SAs (K), respectively, in control or *unp-SV1-MO*-injected embryos at individual nerve segments (control: *n*=12 segments, 6 embryos; *unp-SV1-*MO injection: *n*=9 segments, 5 embryos). (L-Q) Dorsal whole-mount views of SAs (L,N,P) and MEs (M,O,Q) in peroneal nerve (PN) in E12.5 mouse embryo lacking EphA4 in the motor neuron lineage (*Epha4^cko^*): extent of ME projection defects (classified as ranging from I-III) are mirrored by SAs. (R-U) Transverse sections of the sciatic plexus in E12.5 control (R,S) and Ret-deficient (*Ret^ko^*) (T,U) mouse embryos: SA (T) projections mirror severely reduced/absent dorsal ME projections (U) in *Ret^ko^* (asterisk). PN, peroneal nerve; TN, tibial nerve. (V) Correlation of SA and ME extension in mutants with varying ME projection defects at sciatic plexus (mut I-III). (W) Plotting SA against ME pn diameters reveals a linear relationship between extent of SA and ME extension in control, *Epha4^cko^* and *Ret^ko^* embryos (*n*=9, 16 and 17: control, *Epha4^cko^* and *Ret^ko^* embryos, respectively). Scale bars: 20 μm in B,E,H; 200 μm in M,O,Q; 100 μm in S,U.

Do the trajectories chosen by MEs therefore serve as a template for the assembly of peripheral nerves by guiding later-extending axon types? We further tested this by genetically forcing MEs to choose aberrant trajectories, followed by visualizing SAs. In zebrafish, this was achieved by injecting morpholino oligonucleotides targeting a splice variant of the *MUSK* (muscle-specific kinase receptor) gene that were previously established to selectively alter the pattern of ME extension (Zhang et al., 2004). In these larvae, SAs faithfully recapitulated aberrant trajectory choices made by MEs (Fig. 3A-I), with SAs invariably skipping trajectories not occupied by MEs (Fig. 3J,K). In parallel, we took advantage of two previously established gene disruptions in mouse that cause varying degrees of ME misrouting at a binary axon choice point at the base of the limb (supplementary material Fig. S4A-H) (Helmbacher et al., 2000; Kramer et al., 2006; Luria et al., 2008). First, the motor neuron-restricted expression of the receptor tyrosine kinase (RTK) EphA4 was selectively eliminated in the motor neuron lineage through *Cre/loxP* recombination in *Epha4^fx/fx^;Olig2^Cre^* embryos. Second, the expression of the RTK Ret, which is largely motor neuron restricted at the relevant embryonic stages (E9.5-E12), was eliminated in *Ret^−/−^* embryos. Both models selectively affected the dorsal choice of MEs originating from the lateral division of the lateral motor column by altering their responsiveness towards mesenchymal guidance cues (Fig. 3M,O,Q,S,U; supplementary material Fig. S4I-Q) (Helmbacher et al., 2000; Kramer et al., 2006; Luria et al., 2008). As in zebrafish, aberrant ME projections were closely mirrored by SA projections in the mouse hindlimb (Fig. 3L-V; supplementary material Fig. S4I-Q) and revealed a linear relationship between the extent of ME projections and that of the later-extending SAs (Fig. 3W). In both models, SAs thus continued to tightly adhere to MEs, indicating a restriction of EphA4 and Ret function to ME-mesenchyme signaling, but not in SA-ME interactions, during limb innervation. Taken together, SAs thus appear to invariably favor and tightly adhere to trajectories occupied by earlier extending MEs. 

### Sensory axons are dispensable for motor axon guidance

**Fig. 4.**
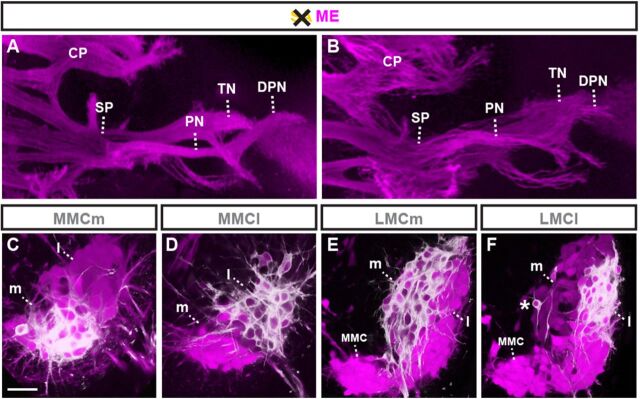
**SAs influence ME fasciculation but not trajectory or target choice.** (A,B) Dorsal whole-mount view: MEs extending into hindlimbs in control (A) and in the absence of SAs in *Wnt1^Cre^;Rosa26^lxstopDTA^* mouse embryos (B). CP, crural plexus; DPN, deep peroneal nerve; PN, peroneal nerve; SP, sciatic plexus; TN, tibial nerve. (C-F) Transverse section of E12.5 thoracic (C,D) and lumbar (E,F) motor columns (magenta indicates *Hb9^MN^::GFP*): retrograde DiI tracing does not detect aberrant ME targeting at the level of columnar divisions. (C) Retrograde DiI tracing from epaxial muscle labels medial division of medial motor column (MMC). (D) Retrograde DiI tracing from hypaxial muscle labels lateral MMC. (E) Retrograde DiI tracing from ventral hindlimb labels *Hb9::eGFP^low^* medial division of lateral motor column (LMC). (F) Retrograde DiI tracing from dorsal hindlimb labels *Hb9^MN^::GFP^high^* lateral LMC (asterisk indicates a possible *Hb9^MN^::GFP^high^* LMCl neuron in the process of lateral migration). Scale bars: 300 μm in A,B; 50 μm in C-F.

We next asked whether these data reflected a hierarchical axon type-dependent relationship by testing whether SAs would conversely influence ME extension by DTA-mediated genetic ablation of DRG neurons in mouse embryos (supplementary material Fig. S5A-F). Consistent with previous data (Huettl et al., 2011), genetic removal of DRG neurons in mouse led to defasciculation of MEs (Fig. 4A,B), which could reflect the loss of repulsive activities exerted by SAs on MEs (Gallarda et al., 2008). As the absence DRG sensory neurons prevents the assembly of DRGs proper, which in turn provide a niche for the expansion of Schwann cell precursors, the defasciculated appearance of MEs could have been alternatively (or additionally) caused by reduced numbers of Schwann cell precursors. At the same time, absence of SAs did not influence the accuracy of trajectory or target selection by MEs (Fig. 4C-F), consistent with MEs normally extending ahead of SAs. SAs thus appear to invariably depend on pre-extending MEs for establishing normally patterned peripheral trajectories, but not vice versa, whereas SAs exert repulsive activities that prevent aberrant intermingling with (and possibly defasciculation of) MEs. 

### Sympathetic axon trajectories are configured by pre-extending sensory and motor axons

**Fig. 5.**
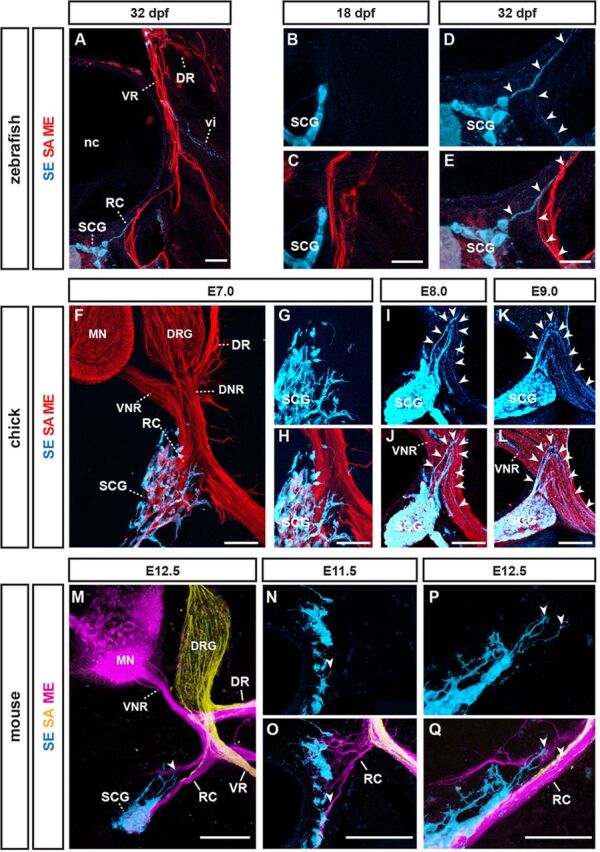
**Conserved late extension of sympathetic efferent axons (SEs).** (A) Transverse section of 32 dpf zebrafish: SEs [blue, anti-tyrosine hydroxylase (TH) immunofluorescence] extending from sympathetic chain ganglion (SCG) along preformed peripheral nerves (red, SA/ME: anti-Tuj1 immunofluorescence). (B,C) TH^+^ SCG neurons prior to initiation SE axon extension at 18 dpf. (D,E) Higher magnifications of A: TH^+^ SEs (arrowheads) extending along preformed peripheral axons. (F) Transverse sections of E7 chick embryo at trunk levels: TH^+^ SCG neurons (blue) around initiation SE extension relative to preformed PNs (red). (G,H) Magnified view of SCG neurons relative to preformed peripheral nerves. (I,J) TH^+^ SE axons begin extending from SCG along PNs at E8 (arrowheads). (K,L) SE axon advancing further peripherally along peripheral nerves at E9 (arrowheads). (M) Transverse section of E12.5 mouse embryo: TH^+^ SEs (arrowhead) beginning to extend from SCGs along pre-extending MEs and SAs in the ramus communicans (RC). (N,O) SCG and rc just prior to initiation of SE extension (arrowhead). (P,Q) Detailed view of SEs (arrowheads) extending along SAs and MEs of RC at E12.5. nc, notochord; DR/VR, dorsal/ventral ramus; vi, intersegmental blood vessel; DNR/VNR, dorsal/ventral nerve roots; RC, ramus communicans. Scale bars: 20 μm in A,C,E; 100 μm in F,H,J,L,M; 50 μm in O,Q.

**Fig. 6.**
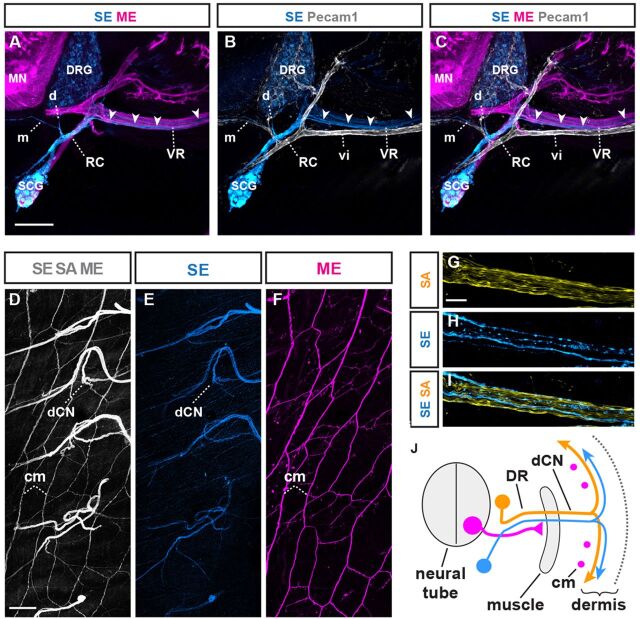
**SEs extend along MEs and SAs to innervate dermis.** (A-C) Transverse section of E14.5 mouse embryo at trunk levels: the majority of trunk-innervating SEs (arrowheads) extend along pre-extending axons (magenta indicates *Hb9::eGFP*) (A,C), rather than intersegmental blood vessels (vi) (gray indicates anti-Pecam1 immunofluorescence) (B,C). Subsets of SEs directly extend medially (m) and dorsally (d) along vasculature (B,C). (D-F) Dorsal whole-mount view of E18.5 mouse: dorsal cutaneous nerve (dCN) axons fanning out in trunk dermis and longitudinal projections by MEs into subdermal *cutaneous maximus* (cm) muscle (D,F). (E) SE projections (blue indicates anti-TH immunofluorescence) through dCN into dermis. (F) ME (magenta indicates *Hb9::eGFP*) innervation of subdermal cm muscle. (G-I) Longitudinal section through dCN at E18.5: separately labeled SEs (blue) and SAs (yellow indicates anti-TrkA immunofluorescence) can be seen. (J) Three axon types in dorsal ramus (ventral ramus, visceral or vascular trajectories are not depicted for simplicity). Magenta dots indicate cross-sections of cm MEs. DR, dorsal ramus; vi, intersegmental blood vessel; RC, ramus communicans; VR, ventral ramus. Scale bars: 150 μm in A; 300 μm in D; 50 μm in G.

**Fig. 7.**
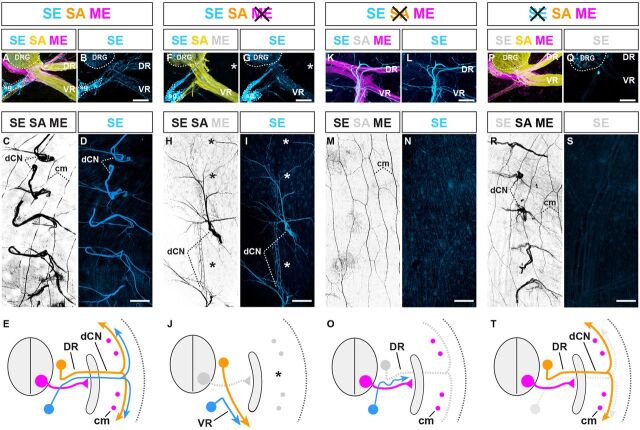
**SE trajectories are configured by pre-extending SAs and MEs.** (A,B) Transverse section of E14.5 mouse embryo: SE (blue), SA (yellow) and ME (magenta) axons extending into dorsal (DR) and ventral (VR) nerve rami. (C) Whole-mount view of dorsal cutaneous nerve (dCN) axons fanning out into trunk dermis. (D) Visualization of SE axons only in same specimen. (E) Summary: three axon types in DR (VR, visceral or vascular trajectories are not depicted for simplicity). Magenta dots indicate cross-sectioned longitudinally projecting *cutaneous maximus* (cm) MEs. (F,G) Loss of dorsal (asterisk) and ventral misrouting of SA projections in the absence of MEs (*Olig^Cre^;Rosa26^fxstopDTA^*) (F) mirrored by SE (G) (asterisk). The converse dorsal misrouting of ventral SAs observed upon ME removal is not shown for simplicity. (H,I) Intermittent loss of dCNs (asterisks) and aberrant pattern of SA projections in the absence of MEs is mirrored by SEs (I). (J) Summary of F-I. (K-N) Initial peripheral extension of SEs along MEs in the absence of SAs (*Adv^Cre^;Isl2^fxstopDTA^*) (note the higher degree of SE fasciculation, compared with control), but failure of SEs to innervate dermis (M,N) (remaining axons in M are subdermal cm MEs). (O) Summary of K-N. (P-Q) Normal appearance of ME, SA trajectories in the absence of SEs (*Dbh^Cre^;Rosa26^fxstopDTA^*). (R,S) Normal appearance of dorsal cutaneous nerves in the absence of SEs. (T) Summary of appearance of P-S. All images are representative of at least five embryos per condition. Scale bars: 100 μm in B,G,L,Q; 300 μm in D,I,N,S. sg, sympathetic ganglion.

Consistent with previous observations (An et al., 2002; Coughlin et al., 1977; Yip, 1990), sympathetic efferent axons (SEs) emerging from sympathetic chain ganglia (SCGs), visualized by tyrosine hydroxylase (TH) immunodetection, were the last axons to extend peripherally in zebrafish (Fig. 5A-E), chick (Fig. 5F-L) and mouse (Fig. 5M-Q), thus recapitulating the phylogenetically late innervation of neural crest-derived autonomic circuits (Butler and Hodos, 2005; Holland, 2009). At trunk levels, SEs emerging from SCGs follow three principal routes to access effector organs (Elfvin, 1983): (1) a minor medial-visceral route; (2) a longitudinal route along sympathetic chain or arteries; and (3) a lateral route adhering to the initial trajectories of MEs and SAs (Fig. 5A,L,M,Q), running parallel to, but not overlapping with, intersegmental blood vessels (Fig. 6A-C; supplementary material Fig. S6E-J) (Nakao and Ishizawa, 1994). In amniotes, the lateral route SEs eventually project along pre-extending cutaneous SAs to innervate dermal glands and smooth muscle as part of the circuits underlying skin thermoregulation (Figs 6D-J and 7C,D) (Elfvin, 1983; Nakao and Ishizawa, 1994). 

**Fig. 8.**
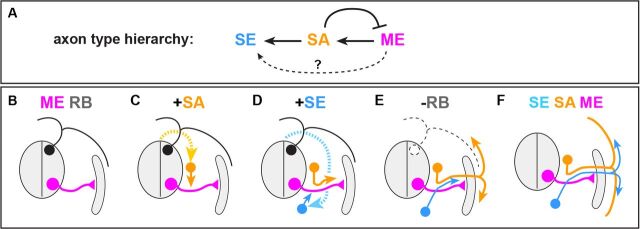
**Conserved axon type hierarchy and ontophyletic model of peripheral nerve assembly.** (A) Hierarchical relationships between the three principal peripheral axon types (whether MEs directly or indirectly influence SEs remains unresolved). (B-F) Ontophyletic model of PN assembly. (B) The phylogenetically oldest MEs extend from MNs in neural tube and actively navigate to peripheral targets guided by mesenchymal cues. RBs contribute to cutaneous escape reflexes prior to emergence of neural crest-derived SAs. (C) SAs use preformed ME trajectories as gateways to peripheral targets. (D) Neural crest-derived SEs subsequently extend peripherally along trajectories established by SAs and MEs. (E) Cutaneous SAs eventually project beyond MEs to innervate dermis, followed by SEs. RBs are lost at the transition to the amniote lineage or degenerate in adult gnathostome anamniotes. (F) Principal pattern of trunk peripheral axon types in a prototypical gnathostome (VR, visceral or vascular trajectories are not depicted for simplicity).

We next tested whether this sequential extension pattern reflected a dependence of SEs on preformed SAs and/or MEs in mouse models that allowed us to separately address the contribution of each of the three principal axon types to peripheral nerve assembly (supplementary material Fig. S6A-D). This was achieved by separately interbreeding the Cre recombination-controlled DTA expression lines *R26^fxDTA^* (Ivanova et al., 2005) or *Isl2^lxstopDTA^* (Yang et al., 2001) with previously established mouse lines that drive Cre expression in motor neuron progenitors (Ivanova et al., 2005), DRG neurons (Hasegawa et al., 2007) or SCG neurons (Parlato et al., 2007) (supplementary material Fig. S6A-D). We found that the aberrant cutaneous SA projection patterns resulting from the absence of MEs (Wang et al., 2011) were precisely mirrored by SEs (Fig. 7F,G), including frequent failure of dermal target innervation by both SEs and SAs (Fig. 7H,I). This suggested that MEs are dispensable for initiating the peripheral extension of SEs, but indirectly influence their trajectory by determining the pattern of SA projections (Fig. 7J). We further tested this by studying SE projections upon selective genetic removal of SAs. In the absence of SAs, SEs extended peripherally along motor projections (Fig. 7K,L), but consistently failed to enter cutaneous trajectories, leaving the trunk dermis entirely devoid of innervation (Fig. 7M-O). At the same time, intersegmental blood vessels developed normally in the absence of MEs and SAs (supplementary material Fig. S6E-J), indicating that failure of cutaneous SE projections upon SA or ME removal was not indirectly caused by impacts on vascular patterning. Last, selective genetic removal of SEs did not result in detectable alterations of peripheral ME or SA projections (Fig. 7P-T), consistent with the initiation of SE extension after most ME and SA trajectories have been established. Thus, although a subset of SEs uses the developing vasculature to access peripheral end organs (Makita et al., 2008), trunk cutaneous SE projections are absolutely reliant on their association with pre-formed SA trajectories that, in turn, are initially guided by MEs (Fig. 8A). 

## DISCUSSION

Our data uncover a conserved hierarchy of axon type-dependent extension and selective fasciculation events governing vertebrate peripheral nerve assembly (Fig. 8A), the temporal order of which recapitulates the successive phylogenetic emergence of peripheral axon types and circuits in the vertebrate lineage (Fig. 8B-F). First, MEs actively navigate to skeletal muscle targets guided by mesenchymal cues (Bonanomi and Pfaff, 2010; Dasen, 2009), thus establishing an initial grid of peripheral trajectories that provides a template for subsequent peripheral nerve assembly (Fig. 8B). Second, SAs use preformed ME trajectories as gateways to their peripheral target organs (Fig. 8C-E). Third, subsets of SEs eventually follow these pre-established trajectories, presumably by responding to cues on SAs (Fig. 8D-F).

MEs extending from cholinergic motor neurons to muscles involved in locomotion represent the sole common feature of peripheral nerves in extant chordates, predating both vertebrates and neural crest-derived circuits (Fig. 8B) (Candiani et al., 2012; Denes et al., 2007; Fetcho, 1992). SAs and SEs, which emerged in agnathostomes and gnathostomes, respectively (Butler and Hodos, 2005; Holland, 2009), were thus from the outset able to rely on pre-evolved axon pathways for accessing peripheral end organs (Fig. 8C-F), This pattern could have been enforced by the segmental remodeling in gnathostomes that brought ventral and dorsal peripheral nerve roots in closer apposition (Butler and Hodos, 2005; Jorgensen, 1998).

The timing of axon extension can profoundly influence the outcome of heterotypic axon-axon encounters (Wang and Marquardt, 2013), which may have been a key factor in preserving peripheral axon type hierarchies from fish to mouse. For example, at least in mouse, the early extension of MEs seems to promote selective fasciculation of SAs with pre-extending MEs by encouraging reverse activation of ephrin A proteins on SA growth cones by their cognate EphA RTKs located on MEs (Wang et al., 2011). In addition, this configuration effectively discourages illicit forward activation of repulsive signaling by the same EphAs on ME growth cones by their cognate ephrin A interaction partners on SAs (Gallarda et al., 2008; Wang et al., 2011), which could otherwise jeopardize the role of MEs in pioneering peripheral nerve tracts. The retention of this hierarchy of axon type-dependent interactions could have been further promoted by the prioritized assembly of ME and RB-based escape reflex arcs over more advanced SA and SE-based somatosensory and autonomic circuits in anamniotes developing through pelagic larval stages (Fig. 8B,C).

Resolving axon type-dependent relationships in the context of the embryo remains challenging, because the underlying signaling mechanisms may operate independently from those determining the responsiveness of axons towards non-axonal guidance cues (Raper and Mason, 2010; Wang and Marquardt, 2013). Thus, altering axon-axon signaling tends to produce changes of axonal trajectories within the confines of relatively fixed pathways that are determined by tissue tracts that are permissive or non-permissive to axon growth. Advancing our understanding of the contribution of axon-axon interactions to nervous system development will therefore depend on expanding the toolkit for unambiguously distinguishing or manipulating the different axon types involved.

Although some of the ontophyletic considerations put forward here presently remain speculative, our findings pave the way for systematically exploring the cellular and molecular basis of the axon-axon interactions contributing to peripheral nerve assembly, and may ultimately serve as a generalized model for how, during nervous system development, phylogenetically newer and older neuron types assemble into common circuitries.

## MATERIALS AND METHODS

### Zebrafish

To label MEs, the previously established transgenic zebrafish line *Tg(NBT:dsRed)* was used, employing a genomic fragment of the *Xenopus laevis* gene *neuronal beta-tubulin* (*NBT*) containing motor neuron regulatory modules to drive red fluorescent protein (dsRed) expression (Blader et al., 2003). To label SAs, the line *Tg(-8.4neurog1:GFP)* was used, which takes advantage of a genomic fragment of the neurogenin 1 gene employing DRG neuron-restricted regulatory elements to drive green fluorescent protein (GFP) expression (Peri and Nusslein-Volhard, 2008). To prevent ME extension, two morpholino oligonucleotides (MO) designed against the Islet1 mRNA [islet1E2-MO (5′-TTAATCTGCGTTACCTGATGTAGTC-3′) and islet1E3-MO (5′-GAATGCAATGCCTACCTGCCATTTG-3′)] were co-injected that have previously been observed to interfere selectively with motor neurogenesis (Hutchinson and Eisen, 2006). To alter, but not prevent, ME extension, we used an MO against the splice variant 1 (SV1) of muscle-specific kinase (MuSK) (unp-SV1-MO: 5′-TATTGTCTTACCTCCATTCTACGGG-3′) that has previously been observed to trigger altered ME navigation (Zhang et al., 2004). MOs were injected at one- to two-cell stage with the following amounts: islet1E2-MO, 3 ng; islet1E3-MO, 3 ng; and *unp-SV1-MO*, 8 ng. All MOs were obtained from Gene Tools (Philomath, OR, USA).

### Chick

Fertilized eggs were staged according to Hamburger and Hamilton (HH) (Hamburger and Hamilton, 1992) and transfected using plasmid injection into the neural tube and *in ovo* electroporation at HH 12/13 as previously described (Marquardt et al., 2005). To label MEs, a previously characterized 4.5 kb promoter fragment of the *Hb9* gene, *Hb9^MN^*, containing motor neuron-specific regulatory elements (Lee et al., 2004) was fused to *mCherry* (Cherry red fluorescent protein with a myristoylation signal ‘m’ for membrane tethering). To co-label SAs, a previously characterized DRG-specific regulatory module of the *Isl1* gene, *Isl1^DRG^*, was fused to a minimal *TATA* box and *mGFP* (m-green fluorescent protein) (Uemura et al., 2005). In addition to DRG neurons, *Isl1^DRG^* labels a subset of Schwann cell precursors (SCPs), presumably originating from their proliferation niche in the DRG (e.g. Fig. 1N, asterisks). For imaging, transverse sections were thus selected containing fewer SCPs to allow unimpeded visualization of SAs. To prevent ME extension, motor neurons were ablated by co-injecting *PGKneolox2DTA* (Addgene: Plasmid 13449) with *Hb9^MN^*::Cre for Cre-mediated activation of DTA expression in motor neurons. After electroporation, eggs were incubated at 38°C until the desired stages.

### Mouse

All mouse work conformed to regulations by the University Medical Center Göttingen animal welfare committee and German animal welfare laws. MEs and SAs were co-labeled by interbreeding the previously established motor neuron-specific marker line *Hb9^MN^::GFP* (Lee et al., 2004) and DRG-neuron marker line *Brn3a^tau:lacZ^* (Trieu et al., 2003). For preventing ME extension, the Cre-controlled ubiquitously expressed diphtheria toxin (DTA) expression line *Rosa26^lxstopDTA^* (Jax stock #006331) was interbred with the motor neuron progenitor (pMN)-specific Cre-driver line *Olig2^Cre^* to selectively ablate pMNs (Ivanova et al., 2005). For preventing SA extension, the neural crest-restricted Cre-deleter line *Wnt1^Cre^* (Jax stock #009107) (Hayashi and McMahon, 2002) was interbred with the Cre-controlled diphtheria toxin (DTA) expression line *Isl2^lxstopDTA^* (Jax stock #007942) (Yang et al., 2001), thus leading to ablation of Isl2^+^ DRG neurons derived from Wnt1^+^ neural crest cells, but not Isl2^+^ motor neurons. To study impacts on SEs, and to prevent potential ablation of Isl2^+^ SCG neurons, the DRG neuron-restricted Cre line *Advillin^Cre^* (Hasegawa et al., 2007) was interbred with *Isl2^lxstopDTA^*. SE extension was prevented by interbreeding *DBH^Cre^* (Parlato et al., 2007) with *Rosa26^lxstopDTA^*. To selectively misroute MEs in the hindlimb, homozygous *Epha4^flox^* (Herrmann et al., 2010) mice were interbred with *Olig2^Cre^* to specifically inactivate *Epha4* in pMNs or embryos homozygous for a *Ret*-null allele (*Ret^ko^*) (Schuchardt et al., 1994) were studied. The mouse lines were genotyped as described previously.

### Immunohistochemistry and imaging

Immunofluorescence staining was performed as described previously (Wang et al., 2011). Primary antibodies used were: rabbit anti-β-galactosidase (Cappel, 1:6000; 55976); goat anti-β-galactosidase (Cappel, 1:12,000; 56028); rabbit anti-GFP (Molecular Probes, 1:2000; AB11122); sheep anti-GFP (Biogenesis, 1:6000; 4745-1051); chicken anti-GFP (Abcam, 1:3000; AB13970); Rabbit anti-dsRed (Clontech, 1:1000; 632496); mouse anti-Tuj-1/βIII-tubulin (Abcam, 1:3000; MMS-435P); rabbit anti-TH (Millipore, 1:1500; 657012); goat anti-TH (Millipore, 1:1500; AB1542); biotin rat anti-mouse PECAM-1 (BD Pharmingen, 1:3000; 553371); rabbit anti-Isl1/2 (gifts from S. L. Pfaff, Salk Institute, USA; 1:3000); Isl1/2 (DSHB, 39.4D5, 1:200); rabbit anti-TrkA (a gift from L. F. Reichardt, UCSF, USA; 1:1000); neurofilament (DSHB, 2H3, 1:200) and neurofilament (DSHB, 4H6, 1:200). Immunofluorescence was detected with Alexa-488, -555 and -637, and streptavidin conjugated secondary antibodies (Molecular Probes, all at 1:1000). Images were collected using Zeiss (LSM 710) or Leica TCS/MP confocal/two-photon microscopes.

### Retrograde tracing

*Hb9::eGFP* transgenic mouse embryos were used to identify motor trajectories. DiI (Sigma)-labeled embryos were incubated in 4% paraformaldehyde overnight at 37°C to permit diffusion, prior to vibratome sectioning at 120 μm for analysis.

### Live imaging of zebrafish

Injected embryos were dechorionated, anesthetized in 0.004% tricaine and mounted in 0.8% low melting agarose on 35 mm glass-bottom dishes. Live imaging was performed at 28°C using a LSM5 Live confocal microscope (Carl Zeiss Microimaging).

### Quantifying the relationship between motor axon and SA extension

For zebrafish larvae, two segments from each were randomly selected for quantification. For each segment, the main branches of motor axon and SA axons were classified in two categories: ‘yes’, indicating motor axon or SA axons project in close association with their counterparts; and ‘no’, indicating motor axon or SA axons extend independently. Next, the percentages of ‘yes’ and ‘no’ among total incidences were calculated for motor axon and SA, respectively. In mouse embryos, the diameters of all motor axons and SAs in peroneal nerves were separately measured at midsection for each embryo.

## Supplementary Material

10.1242/develop.106211_sup1 Supplementary information
