# Supplementary material for: A conserved axon type hierarchy governing peripheral nerve assembly
Source: Development. 2014 May;141(9):dev106211. doi: 10.1242/dev.106211 (PMC13148187; doi:10.1242/dev.106211)
Supplement: Supplementary information [file develop-141-106211-s1.pdf]

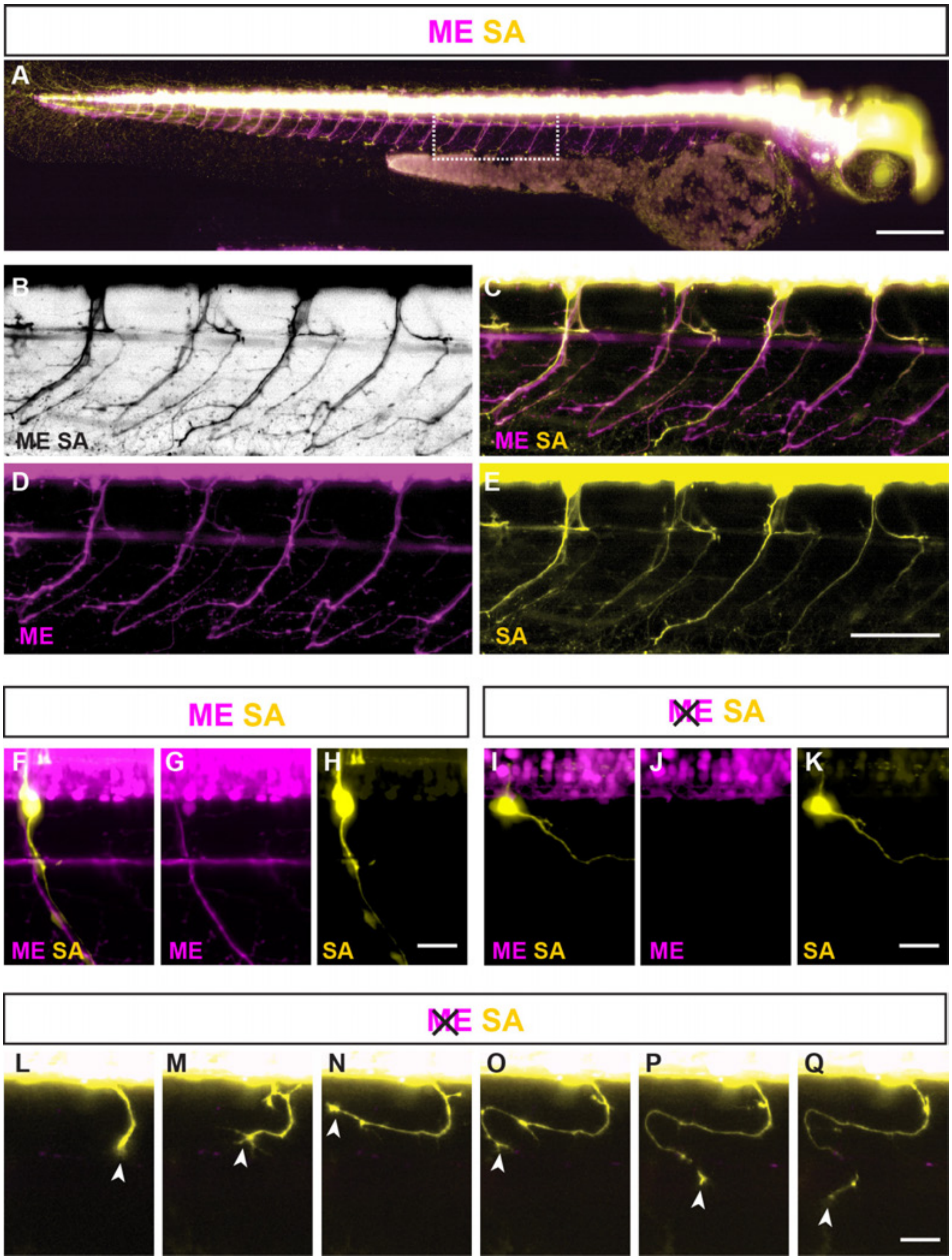


**Fig. S1. Visualizing and manipulating peripheral MEs and SAs in zebrafish.** (A) Lateral wholemount view of peripheral MEs (magenta: *TgNBT:dsRed*) and SAs (yellow: *Tg-8.4neurog1:GFP*) in 72 hpf zebrafish larva. (B-E) Detailed view of trunk PN segments. (B) Monochromatic rendering of all axons. (C) Overlay of ME and SA signals. (D) Separate visualization of MEs. (E) Separate visualization of SAs. (F-H) Example: control SAs and MEs extending from dorsal root ganglion (DRG) neuron and neural tube, respectively. (I-K) Example: aberrant SA extension at segment lacking MEs upon motor neuron ablation by *islet1E2/3-MO* injection. (L-Q) Time-lapse sequence of highly aberrant SA extension in the absence of MEs. Scale bars: 300 µm in A; 50 µm in E; 20 µm in H,K,Q.

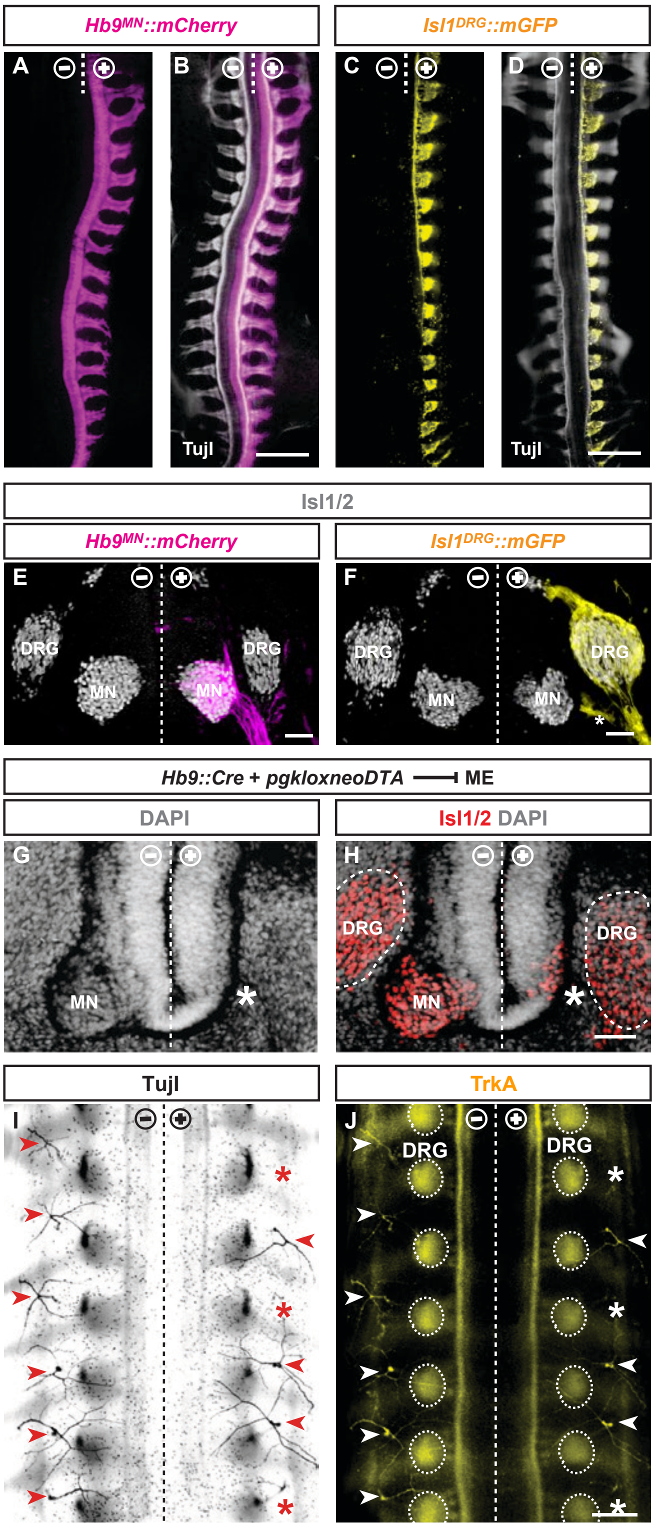


**Fig. S2. Visualizing and manipulating peripheral MEs and SAs in chick.** (A) Dorsal wholemount view of MEs (magenta: *Hb9*$^{MN}$*::mCherry*) in E5 chick embryo (-/+: untransfected/transfected hemitubes). (B) Overlay of MEs with pan-axon label (grey: anti-Tuj-1 immunofluorescence). (C) Dorsal wholemount view of SAs (yellow: *Isl1*$^{DRG}$*::mGFP*) in E5 chick embryo. (D) Overlay of SAs with Tuj-1 immunofluorescence. (E) Transversal section of E6 chick neural tube: unilaterally *Hb9*$^{MN}$*::mCherry*-labelled MEs extending from motor neurons (MNs). Overlay with anti-Isl1 immunofluorescence (grey) to label DRG

neurons and MNs. (F) Transversal section of neural E6 chick tube: *Isl1$^{DRG}$::mGFP*-labeled SAs extending from DRG neurons, in addition to a subset of non-DRG cells (presumably of neural crest origin) also labeled by the *Isl1$^{DRG}$* enhancer (asterisk). (G,H) Transversal section of E5 chick spinal cord: severe reduction of MN numbers, including loss of ventral horn (asterisk), but not DRG neuron numbers, upon unilateral *Hb9$^{MN}$::Cre/PGKneolox2DTA*-mediated MN ablation (grey: cell nuclei labeled by DAPI). (I) Dorsal wholemount view of E7 chick embryo after unilateral *Hb9$^{MN}$::Cre/PGKneolox2DTA*-mediated MN ablation. Innervation of dermis by dorsal cutaneous nerves (arrowheads) visualized by anti-Tuj-1 immunofluorescence (black): intermittent loss of dorsal cutaneous nerves in *Hb9$^{MN}$::Cre/PGKneolox2DTA*-transfected (+), but not control (-) side. (J) Visualization of most DRG neurons and SAs by anti-TrkA immunofluorescence (yellow): loss of dorsal cutaneous SA projections (asterisk), but not DRGs (encircled by dotted lines). Scale bars: 300 μm in B,D,J; 50 μm in E,F,H.

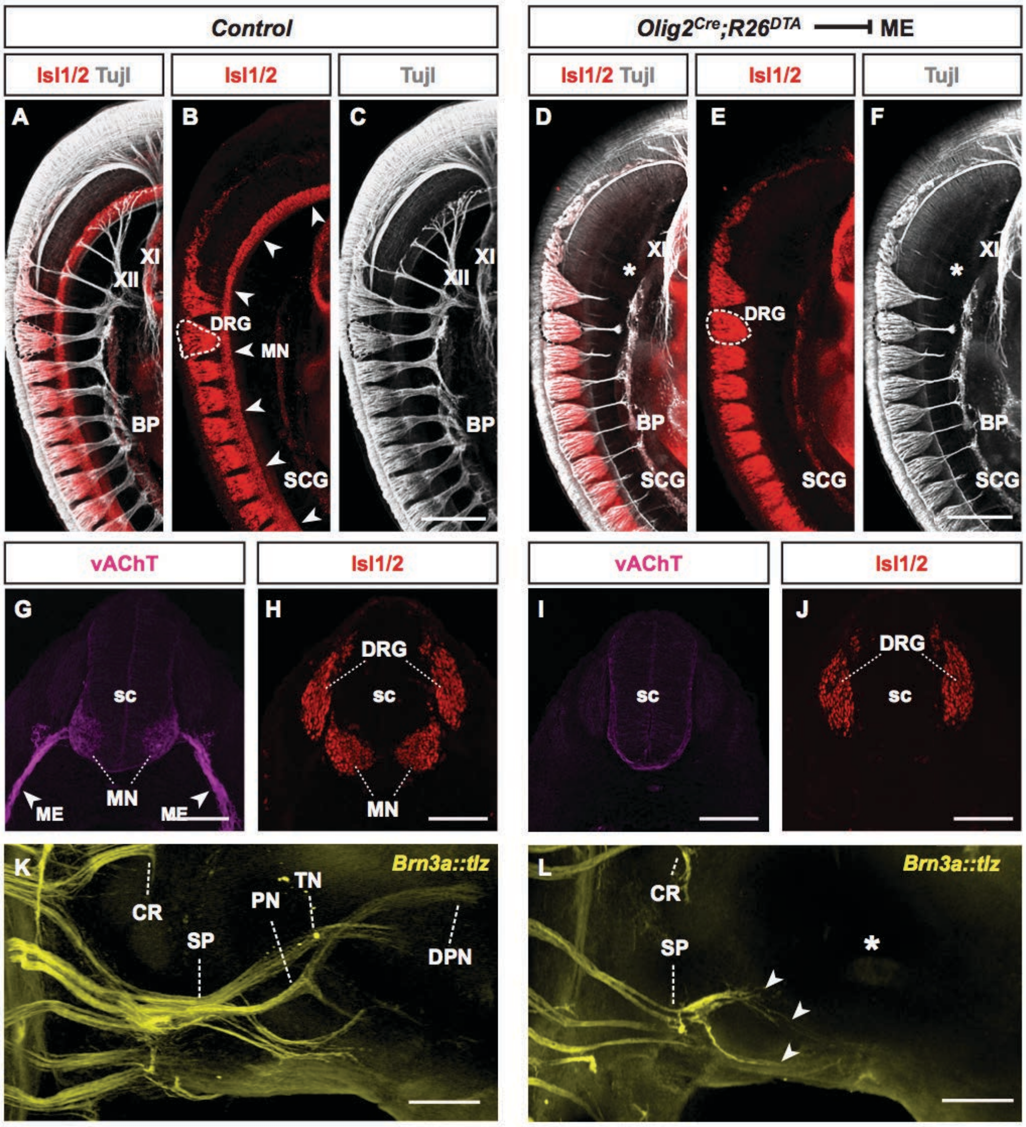


**Fig. S3. Complete and selective ablation of motor neurons in mouse.** (A-C) Lateral wholemount view of E10.5 mouse control embryo (upper thoracic to cranial levels): normal appearance of peripheral axon projections (A,C) (grey: anti-Tuj-1 immunofluorescence). Anti-Isl1/2 immunofluorescence (red) visualizes nuclei of DRG neurons (in ganglia lining the neural tube) and motor neurons (MNs) (arrowheads: motor column within ventral neural tube) (A,B). Abbreviations: SCG (sympathetic chain ganglion neurons), BP (brachial plexus). (D-F) Lateral wholemount view of E10.5 mouse embryo upon MN ablation (*Olig2*$^{Cre}$;*Rosa26*$^{lxstopDTA}$): Complete absence of MNs and pure motor nerves (XII, asterisk) (D,E), but normal appearance of DRGs and sympathetic chain ganglia (scg) (D,F). (G,H) Transversal section of E10.5 control spinal cord (sc): extension of MEs from MNs (magenta: anti-vAChT immunofluorescence) (G) and presence of DRG neurons and MNs (red: anti-Isl1/2 immunofluorescence) (H). (I,J) Transversal section of E10.5 *Olig2*$^{Cre}$;*Rosa26*$^{lxstopDTA}$ spinal cord: complete absence of MEs (I, J), but not DRG neurons (J). (K) Dorsal wholemount view of SAs (yellow: *Brn3a*$^{tlz}$) at sciatic plexus (SP) in E12.5 mouse. Abbreviations: CP (crural plexus), DPN (deep peroneal nerve), PN (peroneal nerve), TN (tibial nerve). (L) Near-total failure of SAs (arrowheads) to innervate limb beyond plexus in the absence of MEs in *Olig2*$^{Cre}$;*Rosa26*$^{lxstopDTA}$ embryo (asterisk). Scale bars: 200 µm in C,F,G,H,I,J,K,L.

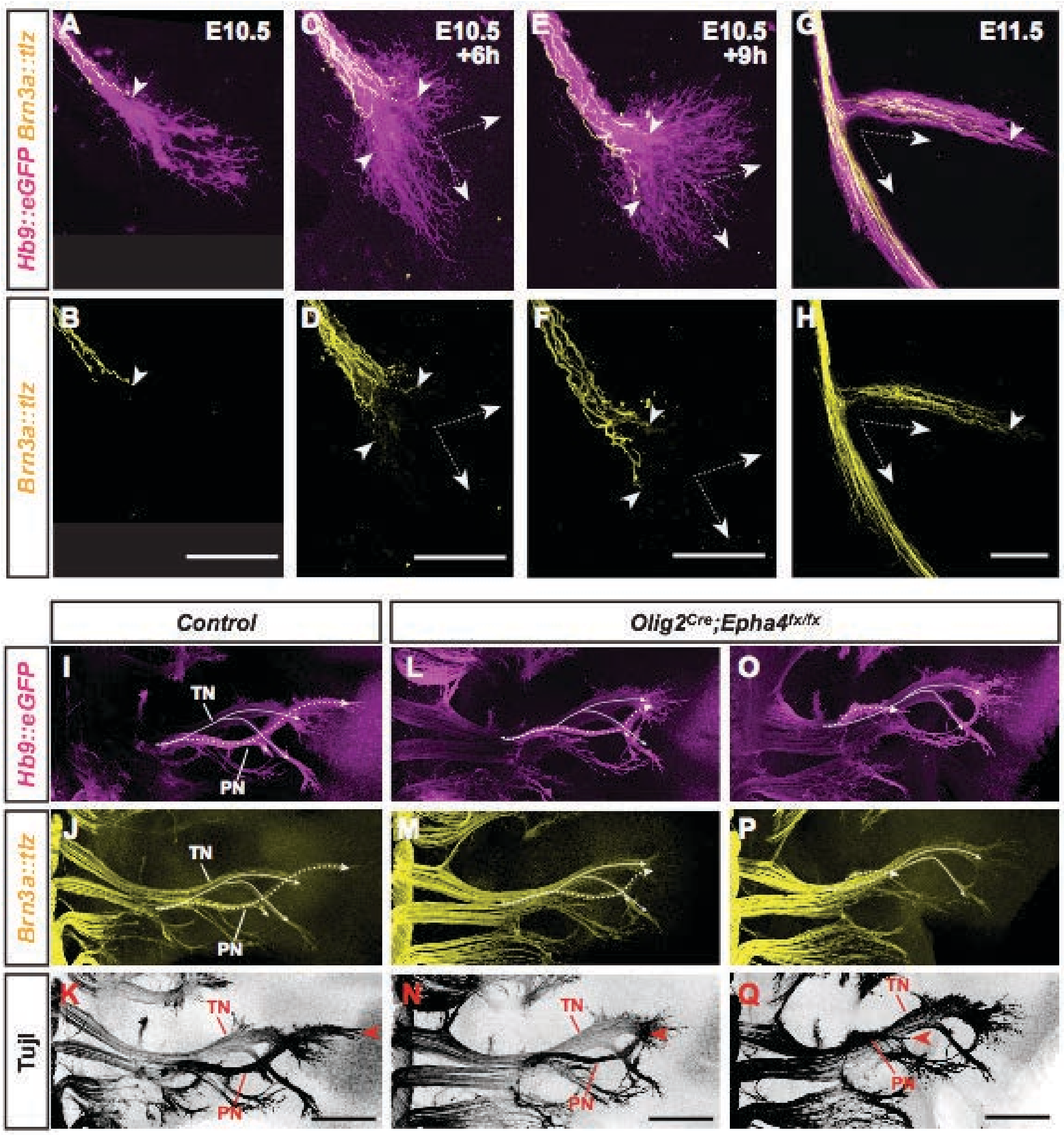


**Fig. S4. Visualizing ME and SA extension and selectively manipulating ME extension in mouse hindlimb.** (A-H) Sequential extension of MEs (magenta) and SAs (yellow) beyond the sciatic plexus during development. Arrowheads: forefront SA growth cones extending along pre-extending MEs. Arrows (C-H): routing of MEs and SAs along dorsal and ventral nerve sheets. (I-Q) Dorsal wholemount view of MEs (I,L,O), SAs (J,M,P) or all axons (K, N,Q) in control (I-K) and *Olig2*$^{Cre}$;*Epha4*$^{fx/fx}$ mouse embryos (L-Q) at E12.5. Dotted lines trace peroneal (PN) and solid lines tibial (TN) nerves. (J-P) Relative severity of ventral misrouting of MEs in *Olig2*$^{Cre}$;*Epha4*$^{fx/fx}$ hindlimb (L,O) is faithfully mirrored by reduced/lost dorsal SA extension (M,P), also apparent by corresponding reduction/loss of pan-axon label (N,Q). Scale bars: 100 µm in B,D,F,H; 300 µm in K,N,Q.

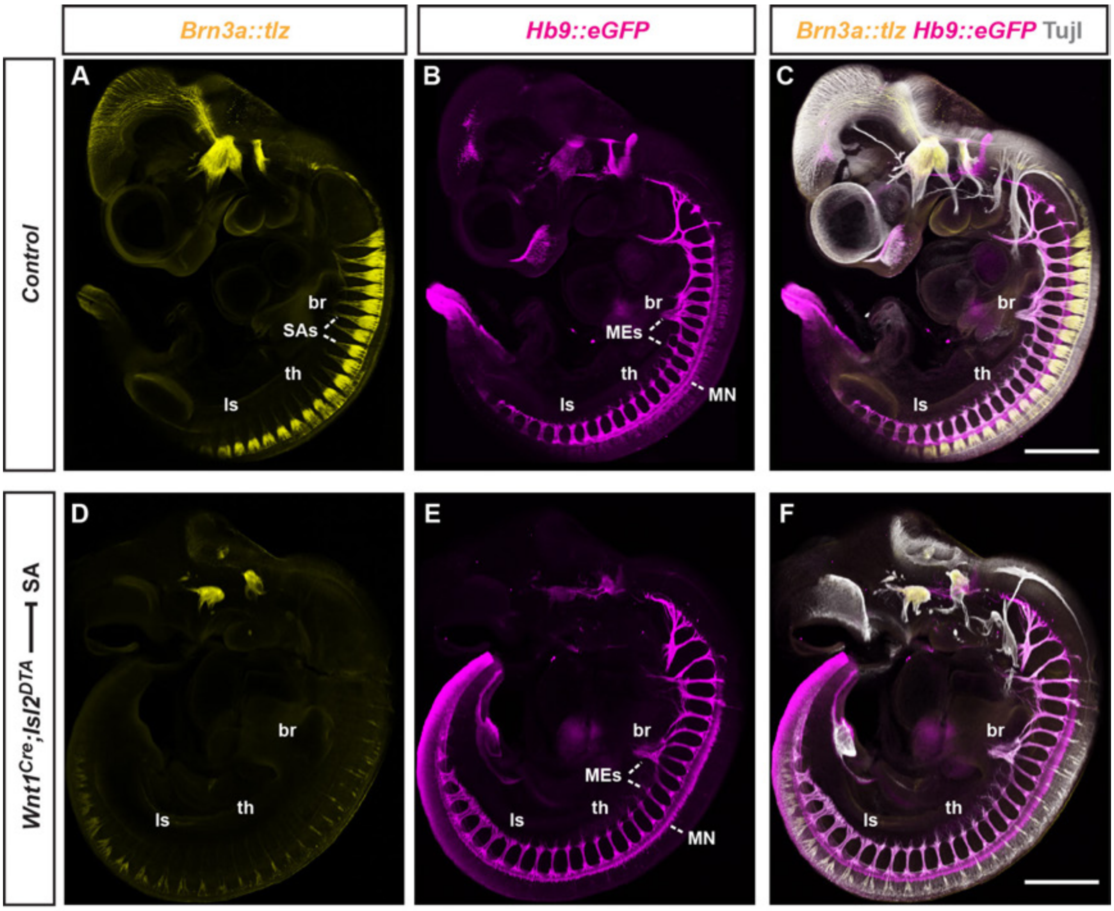


**Fig. S5. Selective ablation of DRG neurons and SAs in mouse.** (A-C) Lateral wholemount view of E10.5 mouse control embryo: normal appearance of peripheral SAs (yellow: $Brn3a^{tlz}$), MEs (magenta: $Hb9^{MN}$*::GFP*) and pan-axon label (grey: anti-Tuj1 immunofluorescence). (D-F) Near-absence of DRG neurons (D,F), but not MNs or MEs (E,F) in $Wnt1^{Cre}$;$Isl2^{lxstopDTA}$ embryo. Note: aberrant head structures, likely due to cranial neural crest defects. Abbreviations: br (brachial), th (thoracic), ls (lumbosacral).

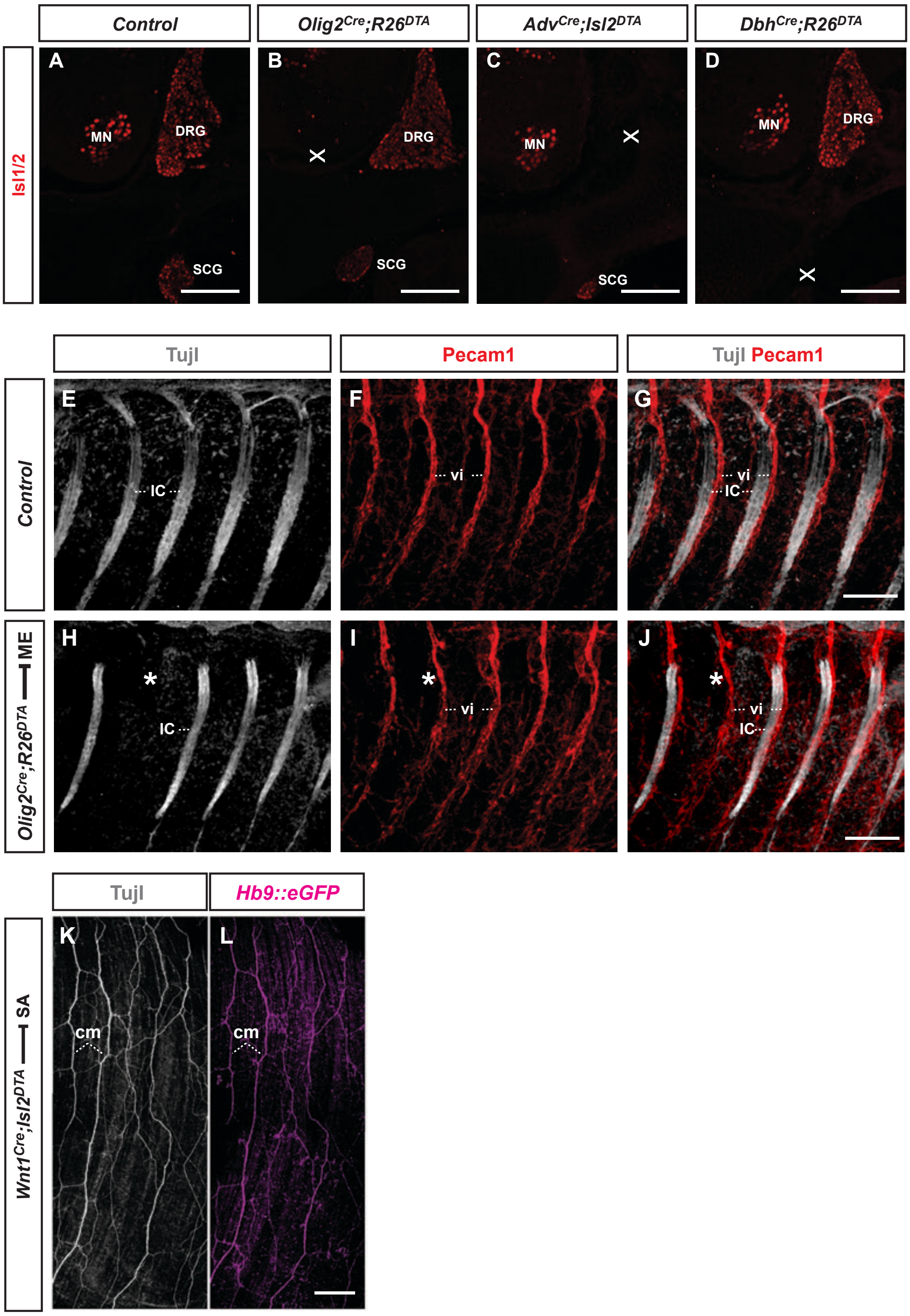


**Fig. S6. Selective manipulation of peripherally projecting neuron types in mouse and relationship between PNs and vasculature.** (A-D) Transversal section of E14.5 mouse embryos at trunk levels: anti-Isl1/2 immunofluorescence visualizes nuclei of motor neurons (MNs) in spinal cord, DRG and SCG neurons in control embryo (A), selective ablation of MNs in *Olig2*$^{Cre}$;*Rosa26*$^{lxstopDTA}$ (B), selective ablation of DRG neurons in *Adv*$^{Cre}$;*Isl2*$^{lxstopDTA}$ (C), selective ablation of SCG neurons in *Dbh*$^{Cre}$;*Rosa26*$^{lxstopDTA}$ (D). (E-G) Lateral wholemount view of E14.5 mouse trunk: spatial arrangement of intercostal PNs (IC, grey) (E,G) and intersegmental blood vessels (vi, red) (F,G). (H-J) Lateral wholemount view of E14.5 trunk in *Olig2*$^{Cre}$;*Rosa26*$^{lxstopDTA}$ mouse embryo: intermittent absence of intercostal

nerves (IC) (H,J) does not affect formation of intersegmental blood vessels (vi) (I,J). (K-L) Dorsal wholemeount view of *Adv*$^{Cre}$;*Isl2*$^{lxstopDTA}$ E18.5 mouse embryo: subdermal cutaneous maximus (cm) ME projections labeled by pan-axon marker anti-Tuj-1/βIII-tubulin (grey) and ME marker *Hb9*$^{MN}$*::eGFP*. Scale bars: 150 μm in A-D; 300 μm in G,J,L.
